# A Secure Online Fingerprint Authentication System for Industrial IoT Devices over 5G Networks

**DOI:** 10.3390/s22197609

**Published:** 2022-10-07

**Authors:** Aseel Bedari, Song Wang, Wencheng Yang

**Affiliations:** 1 Department of Engineering, La Trobe University, Bundoora, VIC 3086, Australia; 2 School of Mathematics, Physics and Computing, University of Southern Queensland, Toowoomba, QLD 4350, Australia

**Keywords:** fingerprint authentication, secure authentication, cancelable fingerprint template, IIoT, 5G network

## Abstract

The development of 5G networks has rapidly increased the use of Industrial Internet of Things (IIoT) devices for control, monitoring, and processing purposes. Biometric-based user authentication can prevent unauthorized access to IIoT devices, thereby safeguarding data security during production. However, most biometric authentication systems in the IIoT have no template protection, thus risking raw biometric data stored as templates in central databases or IIoT devices. Moreover, traditional biometric authentication faces slow, limited database holding capacity and data transmission problems. To address these issues, in this paper we propose a secure online fingerprint authentication system for IIoT devices over 5G networks. The core of the proposed system is the design of a cancelable fingerprint template, which protects original minutia features and provides privacy and security guarantee for both entity users and the message content transmitted between IIoT devices and the cloud server via 5G networks.Compared with state-of-the-art methods, the proposed authentication system shows competitive performance on six public fingerprint databases, while saving computational costs and achieving fast online matching.

## 1. Introduction

With the development of 5G mobile communication networks, industrial automation is expanding at a rapid pace. This transition is also known as the “Industrial Internet of Things (IIoT)” or “smart factories” [1]. 5G networks have a host of desirable features, such as high transmission rate, low latency, low energy consumption and massive machine-type communication [1]. The development of 5G networks greatly increases the use of IoT/IIoT devices for control, monitoring and processing purposes. Therefore, industries such as manufacturing are utilising the IIoT to make machines more intelligent [2]. Despite the benefits of the 5G reflected by IIoT applications, data security is a critical challenge. For example, a manufacturer can collect raw data from its devices (e.g., sensors) and even assets. These data may contain private information, such as customers’ personal information (e.g., their behavior characteristics) [3], causing information leakage and privacy invasion. Hence, appropriate security measures must be taken to ensure data safety and integrity in the IIoT.

User authentication is key to reliable IIoT applications like advanced manufacturing [4], as it prevents unauthorized or fraudulent access to IIoT devices. A secure authentication system can guarantee that data transfer is between legitimate IIoT users and devices or equipment. Compared with password-based authentication, biometric systems offer practical convenience and enhanced authentication performance. Fingerprint recognition is one of the most established biometric authentication methods, with strong feature extraction and high recognition accuracy [5]. However, raw fingerprint data, stored as templates in central databases or on IoT/IIoT devices, if left unprotected, can be at risk, because everybody’s fingerprint is unique and permanent. Once a raw fingerprint template is stolen, it is lost forever and cannot be re-issued, replaced or destroyed. Fraudulent use of stolen fingerprint template data and privacy invasion make template protection a crucial task in IoT/IIoT-related applications. Unfortunately, most fingerprint authentication systems currently used in the IoT/IIoT do not have template protection [6], endangering the security of raw fingerprint data. Moreover, traditional biometric authentication faces slow, limited database holding capacity and data transmission problems [1].

**Figure 1:**
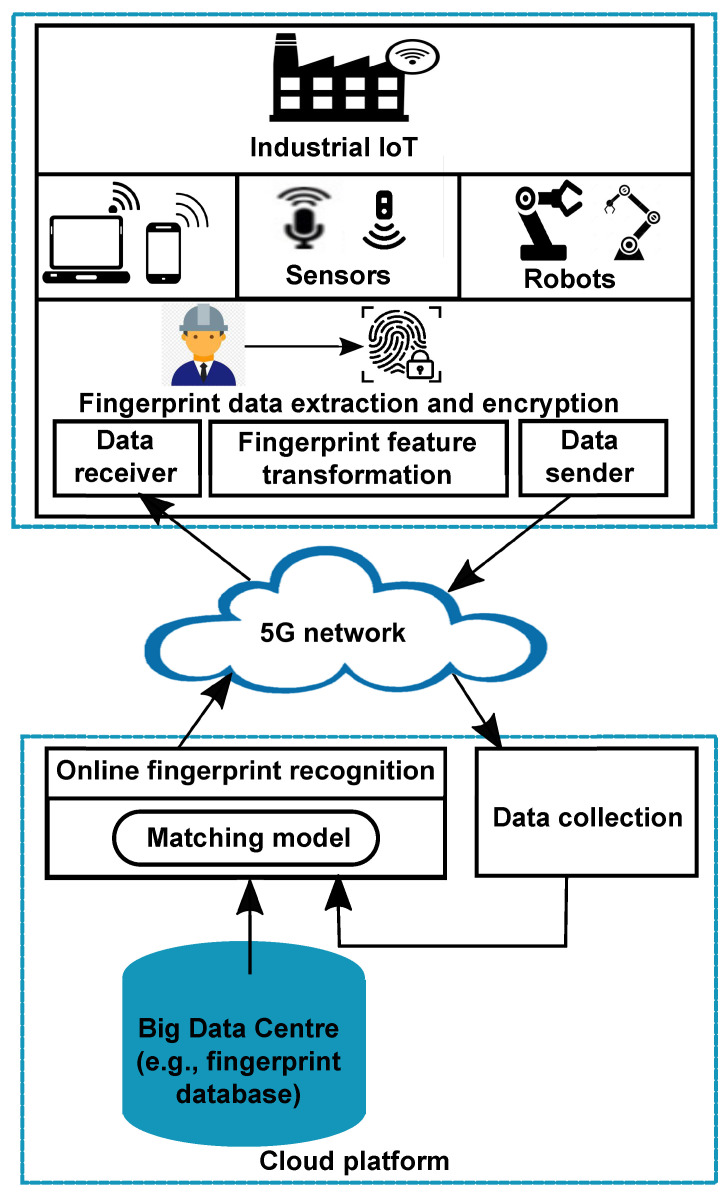
Architecture of the proposed online fingerprint authentication system.

To address the above issues, in this paper we propose a secure online fingerprint authentication system for IIoT devices over 5G networks. The proposed system features the design of a cancelable fingerprint template, which protects original fingerprint minutia features and therefore provides privacy and security guarantee. To take advantage of 5G’s high-speed transmission and the cloud’s powerful processing capability, the proposed authentication system carries out online fingerprint matching in the encrypted domain in the cloud server. Compared with state-of-the-art methods, the proposed authentication system shows competitive performance on six public fingerprint databases, while saving computational costs and achieving fast online matching. Figure 1 shows the architecture of the proposed fingerprint authentication system. Note that the proposed system is not confined to 5G networks because the architecture of our system suits both 5G and non-5G networks. It is worth mentioning that the desired features of 5G networks improve the speed of online matching, thus increasing the efficiency of the proposed system.

The contributions of this paper are summarized as follows:

1. The proposed online fingerprint authentication system is equipped with template protection. If a stored template is compromised, raw fingerprint data cannot be retrieved and the compromised template can be revoked and replaced with a new one. Thus, data security is heightened in the access control of IIoT devices.
2. The proposed authentication system is highly efficient, evidenced by reduced template size, low computational costs and fast online matching, making it suitable for IIoT-related applications.
3. Not only does the recognition accuracy of the proposed system with template protection level with the baseline unprotected system, but it also outperforms state-of-the-art fingerprint authentication systems with template protection. The strong performance of the proposed system ensures that critical and/or sensitive data in the IIoT environment are only accessed by genuine (i.e., authorized) users.

The rest of this paper is organised as follows. Section 2 presents the related work of biometric authentication in the IoT and cancelable fingerprint templates. Section 3 details the proposed secure online fingerprint authentication system. The experiment results are reported and discussed in Section 4. The conclusion is given in Section 5.

## 2. Related Work

### 2.1. Biometric Authentication in the IoT

Liu et al. [1] proposed an online biometric authentication structure for future 5G communication systems. The authors argued that the security level of the internet can be improved if biological characteristic authentication is applied, such as fingerprint identification. Wilkins [4] reviewed how biometrics can be utilized to better secure manufacturing protocols and processes. The author discussed about the options of choosing correct technologies to improve safety and security in factories. Since hackers continue to find new ways to gain information, the author stated that it is necessary to make systems adaptable, as security is a moving objective. Yang et al. [7] developed a privacy-preserving lightweight fingerprint authentication system for resource-limited IoT devices. The proposed method uses a block logic-based algorithm to minimise the template size and achieve good performance.

Ayub et al. [8] introduced a lightweight secure three-factor biometric-based authentication protocol for e-Healthcare applications in the IoT through the 5G technology. The proposed protocol is cost-effective in terms of computational and communication costs compared to many existing e-Health cloud authentication protocols. Security analyses show that the protocol can resist attacks such as user anonymity, offline password guessing, impersonation and stolen smartcard attacks, but is vulnerable to man-in-the-middle and replay attacks [9].

Sedik et al. [10] used deep learning models, implemented with a dataset of fingerprint images, to detect alterations to biometric modalities and discriminate pristine, adulterated and fake biometrics in 5G-based smart cities. The proposed scheme computes the probability of a biometric which is tampered with. Bedari et al. [11] proposed a two-stage feature transformation-based fingerprint authentication system to protect user privacy in the IoT. The authors designed a weight-based fusion mechanism in the first stage, and a non-invertible transformation in the second stage. With strong performance and energy efficiency, the proposed system is suited to resource-constrained IoT devices.

### 2.2. Cancelable Fingerprint Templates

Cancelable biometrics is an important biomtric template protection technique. It converts original biometric feature data non-invertibly to a ‘distorted’ version through a one-way transformation. When the stored (transformed) template is compromised, it can be easily revoked and replaced with a new template by changing the user-specific key.

Yang et al. [12] proposed a feature-adaptive random projection-based cancelable fingerprint authentication system. The proposed feature-adaptive random projection can mitigate the negative effect of biometric uncertainty on matching performance. Shahzad et al. [13] designed fingerprint cancelable templates with dual protection to improve security. The dual protection involves the window-shift-XOR model and partial discrete wavelet transform. Bedari et al. [14] developed cancelable fingerprint templates using binary features of the minutia cylinder-code (MCC) [5] based on a dynamic random key model, named Dyno-key model. The Dyno-key model uses randomly generated keys to dynamically select elements from MCC’s binary feature vectors so that uncertainty is added to the generated cancelable template.

Jin et al. [15] proposed cancelable fingerprint templates by transforming real-valued feature vectors [16] into a ranking-based representation using locality sensitive hashing, called index-of-max (IoM) hashing. Later, Kim et al. [17] built on the IoM hashing method in [15] and derived sparse combined index-of-max (SC-IoM) hashing. The SC-IoM method extracts the largest and second largest indices of user-specific randomly projected features and uses a large number of hash functions to obtain satisfactory authentication performance. Abdullahi et al. [18] introduced a cancelable fingerprint hash scheme based on the Fourier-Mellin transform and fractal coding. The proposed method performs well over fingerprint databases of reasonably good image quality, but its performance deteriorates when the quality of fingerprint images is poor.

Li et al. [19] designed Indexing-Min-Max (IMM) hashing-based cancelable fingerprint templates. The proposed method embeds the explicit fixed-length fingerprint feature vector non-linearly into the implicit ordering space. Unlike the original IoM hashing method, the IMM hashing model collects implicit indices of the maximum and minimum values from multiple random tokenized partial Hadamard transforms to enhance security and recognition accuracy simultaneously. Li and Wang [20] proposed a one-factor cancelable fingerprint authentication scheme based on the novel minimum hash signature (NMHS) and secure extended feature vector (SEFV). The NMHS algorithm produces the hash codes of binary fingerprint templates and utilises the XOR operation in the hashing process to improve stability. The SEFV is then used to obtain a pseudo identifier. In the authentication stage, the pseudo identifier is produced with genuine queries using the auxiliary data provided by the system. A fusion rule is introduced to enhance authentication performance.

Li et al. [21] presented a cancelable fingerprint binary code generation scheme based on one permutation hashing. The transformed feature is bit-wise uniformly distributed and offers fast matching. The proposed method also applies the partial Haar transform to strengthen the security of the cancelable fingerprint template. Lee et al. [22] proposed a one-factor cancelable fingerprint template, called extended feature vector (EFV) hashing. The EFV hashing utilizes a permutation key separate from the fingerprint feature data to yield the cancelable template. With XOR encryption, the key is not stored in its original form. Yang et al. [23] designed a linear convolution-based cancelable fingerprint authentication system. The proposed system uses a help vector as the second input to the linear convolution function so as to increase the immunity of resultant templates to errors from the feature data. To protect the help vector, the authors developed a feature-guided index generation method. Despite robust security, the proposed system does not perform well over databases with poor image quality.

A comparison of the above literature is shown in Table 1. It is clear from Table 1 that the existing cancelable fingerprint templates have a limitation on striking a balance between providing strong security and attaining good authentication performance over databases of low image quality. In addition, energy-efficient data storage and low computational costs are critical when designing secure biometric authentication systems for the IoT/IIoT. Hence, more effort is needed in developing lightweight schemes that will reach a balanced trade-off. To this end, in this paper we design a secure online fingerprint authentication system whose core component is a cancelable template for the protection of original fingerprint data. The proposed system not only achieves satisfactory performance over databases with poor-quality images, but it is also efficient with size-reduced templates and fast online matching.

## 3. Proposed System

**Figure 2:**
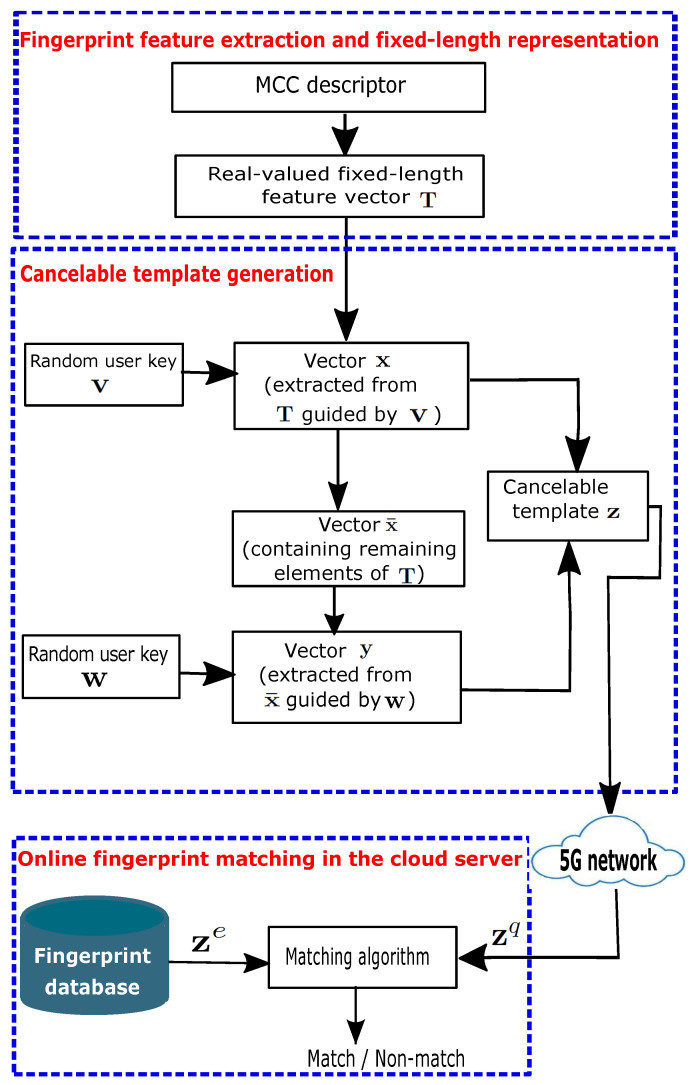
Block diagram of the proposed authentication system.

In this section, we propose a secure online fingerprint authentication system for IIoT devices over 5G networks. The core of the proposed system is the design of a cancelable fingerprint template. Figure 2 illustrates the block diagram of the proposed authentication system, which is made up of three steps. The first step is fingerprint feature extraction and representation, where a real-valued, fixed-length feature vector is extracted based on the method in [16]. Since this fixed-length feature vector contains the original minutia information, to protect it, we develop a cancelable fingerprint template in the second step. In the third step, the protected template is transmitted over the 5G network and online fingerprint matching is conducted in the cloud server.

### 3.1. Fingerprint Feature Extraction and Fixed-Length Representation

The well-known fingerprint minutia descriptor, MCC [5] is one of the best performing minutia-based feature extraction methods. The MCC discretizes the neighborhood of each minutia into a three-dimensional cell structure, called cylinder. Each of MCC’s cylinders is represented by a variable-sized binary feature vector. To improve matching speed and the applicability of variable-sized representations in biocryptography-related applications, Jin et al. [16] refined the MCC and proposed fixed-length feature representations. Through kernel learning and point-to-string conversion [16], a real-valued, fixed-length feature vector is derived from MCC’s variable-sized binary features. Let us denote the fixed-length real vector by T, expressed as

(1) T=[T(1),T(2),⋯,T(m)]

The derivation of T in (1) is detailed in [16]. Vector T is real-valued with length *m*, serving as the input to the proposed cancelable template generation algorithm in the next section.

### 3.2. Cancelable Template Generation

The fixed-length real vector T is unprotected. As it contains the original fingerprint minutia features, there would be serious security and privacy issues (e.g., privacy invasion), if T is compromised. That is why we design a cancelable fingerprint template to protect vector T.

First, we generate two random vectors v and w, both of length *n*, written as

(2) $$v=[v_{1},v_{2},\cdots ,v_{n}]$$

(3) $$w=[w_{1},w_{2},\cdots ,w_{n}]$$

where $1<n\leq \frac{m}{2}$, and all entries of v and w are strictly positive integers with $v_{i}\neq v_{j}$ and $w_{p}\neq w_{q}$, for all i≠j and all p≠q. Random vectors v and w serve as index vectors.

Next, we use v to extract elements from vector T in (1) whose indices are of the same values as entries in v. This forms a new vector x of length *n*, i.e.,

(4) $$\begin{array}{cc} x & =[x(1),x(2),\cdots ,x(n)]\\ \; & =[T\left(v_{1}\right),T\left(v_{2}\right),\cdots ,T\left(v_{n}\right)] \end{array}$$

The remaining elements of the vector T are put in a vector, called $\bar{x}$, whose length is m−n. The vector $\bar{x}$ can be expressed as

(5) $$\bar{x}=\left[\bar{x}\left(1\right),\bar{x}\left(2\right),\cdots ,\bar{x}\left(m-n\right)\right]$$

Then, similar to the handling of vector v, we use the index vector w in (3) to extract *n* elements from vector $\bar{x}$ in (5) whose indices coincide with entries of w. Thus, we obtain a new vector y of length *n*. That is,

(6) $$\begin{array}{cc} y & =[y(1),y(2),\cdots ,y(n)]\\ \; & =[\bar{x}\left(w_{1}\right),\bar{x}\left(w_{2}\right),\cdots ,\bar{x}\left(w_{n}\right)] \end{array}$$

Finally, we find the element-wise average of vectors x and y. Hence, we have

(7) $$z=\frac{1}{2}\left(x+y\right)$$

The real vector z=[z(1),z(2),⋯,z(n)] is the resultant cancelable template.

The above process transforms the fixed-length real vector T in (1) to the vector z in (7). This transformation is non-invertible, because it realizes a many-to-one mapping. From the vector z, it is highly unlikely to recover the elements of vectors x and y (see the detailed analysis in Section 4.5). Random vectors v and w are the parameter keys in the proposed method. This means that different v and w result in different transformed templates z, thus making z revocable.

The transformed template z provides strong protection to the original MCC features contained in the fixed-length real vector T. This protection can be justified in two aspects. First, vector T cannot be retrieved even if the transformed template z and user-specific keys v and w are all compromised (see security analysis in Section 4.5). Second, if the transformed template z is compromised, a new template can be issued simply by changing the user keys v and w. The new template is unrelated to the compromised template and can be generated differently from one application to another (see unlinkability analysis in Section 4.4).

The proposed cancelable template design is efficient in that its implementation does not involve any complex computations or time-consuming iterations. The user key generation and cancelable template design can be performed efficiently on IIoT devices.

The size of the transformed template z is less than half of the length of the original feature vector T. This is because the length of z is *n* and $1<n\leq \frac{m}{2}$ with *m* being the length of T. Therefore, the proposed authentication system attains substantial savings in memory storage, while achieving high recognition accuracy (see the experiment results and analysis in Section 4). Such savings are beneficial to IIoT devices and also reduce matching time (see Analysis of Computational Costs in Section 4.2).

### 3.3. Online Fingerprint Matching in the Cloud Server

There are usually two stages in biometric authentication—the enrollment stage and the verification stage, which are applicable to online fingerprint authentication in the IIoT. Specifically, in the enrollment stage, the transformed template $z^{e}$, where the superscript *e* denotes ‘enrolled’, is generated by following the process in Section 3.2. The transformed template $z^{e}$ is stored in a fingerprint database in the cloud’s big data centre. In the verification stage, a user (i.e., the query) intending to access an IIoT device goes through the same procedure in Section 3.2 to generate the query’s (transformed) template $z^{q}$, where the superscript *q* denotes ‘query’. Since $z^{q}$ is the transformed template of the query, it is protected and should be secure when transmitted through a 5G network to the cloud server, where fingerprint matching is conducted. Thus, to make use of 5G networks’ super-fast transmission and the cloud server’s powerful processing ability, we match the enrolled and query templates online in the cloud server. Note that both the enrolled and query templates are transformed versions of the raw templates. In other words, fingerprint matching is conducted in the transformed or encrypted domain, so no disclosure of raw fingerprint data would occur in the matching process. Moreover, since only the transformed templates rather than the original are transmitted via the 5G network, there should be no security concern about leaking or revealing original fingerprint features.

The similarity score $S(z^{e},z^{q})$ between the (transformed) enrolled and query templates is calculated as

(8) $$S\left(z^{e},z^{q}\right)=\frac{\sum_{k=1}^{n}\left(z^{e}\left(k\right)*z^{q}\left(k\right)\right)}{∥z^{e}{∥}_{2}^{2}+{∥z^{q}∥}_{2}^{2}}$$

where ${∥\cdot ∥}_{2}$ is the $l_{2}$ norm of vectors. The above equation is adapted from Equation (32) in [16].

The similarity score $S(z^{e},z^{q})$ ranges from 0 to 1 with 0 meaning that there is no similarity between the enrolled template and the query, and 1 meaning that the query is completely similar to the enrolled template. The higher the score $S(z^{e},z^{q})$, the more similar the query is to the enrolled template. Therefore, the user is given access to the IIoT device or application only if the similarity score is higher than a predefined threshold.

## 4. Experiment Results and Analysis

To evaluate the proposed authentication system, we conduct extensive experiments over six public fingerprint databases—FVC2002 DB1-DB3 [24] and FVC2004 DB1-DB3 [25]. Each database consists of 800 fingerprint images with varying qualities, and these fingerprint images were collected from 100 users with 8 impressions per user. In accordance with the requirements of cancelable biometrics, the designed authentication system is evaluated on whether it satisfies performance preservation after transformation, revocability and diversity, unlinkability and non-invertibility.

The commercial fingerprint recognition software VeriFinger SDK [26] is employed to extract minutiae from fingerprint images in the aforementioned databases. The performance of the proposed authentication system is evaluated based on three performance indices—False Acceptance Rate (FAR), False Rejection Rate (FRR) and Equal Error Rate (EER). The FAR represents the probability of mistaking two fingerprints from different entities to be from the same entity, while the FRR means the probability of mistaking two fingerprints from the same entity to be from different entities. The relationship between the Genuine Acceptance Rate (GAR) and the FRR is GAR+FRR=1. The EER is the error rate when the FAR is equal to the FRR.

The performance indices described above are obtained from genuine testing and imposter testing. Genuine testing is conducted by matching two impressions from the same finger, whereas imposter testing is performed by matching two impressions from different fingers. For each database, according to the method in [16], the first to third impressions of each finger are used as the training samples to generate the real-valued, fixed-length feature vector T in (1), and the remaining impressions (i.e., fourth to eighth) of each finger are used for testing purposes. In our experiments, vector T in (1) acts as the input to the proposed cancelable generation algorithm. The original FVC protocol [27] is adopted in our experiments.

### 4.1. Performance Evaluation

The performance of the proposed scheme is evaluated under the worse-case scenario, where the user-specific key is lost. We simulated this worst case (i.e., the lost-key scenario) by assigning all the users in a database the same key, namely the same random vector v in (2) and the same random vector w in (3).

**Figure 3:**
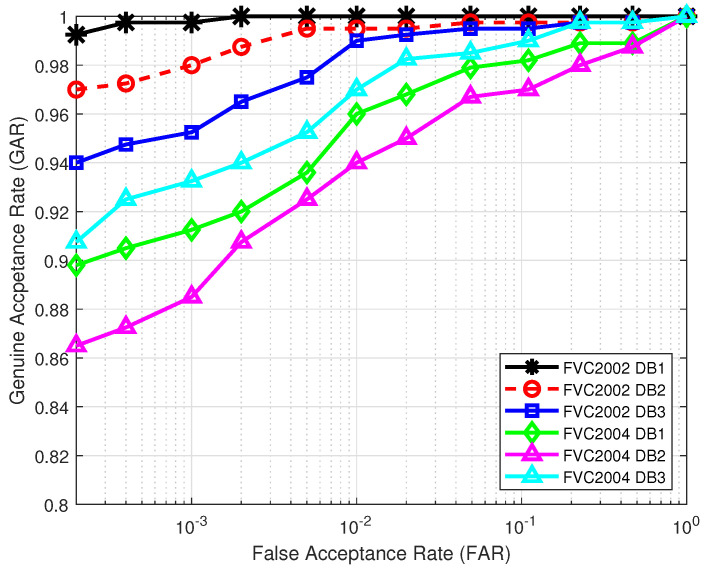
ROC curves in the lost-key scenario under the original FVC protocol.

First, we plot the receiver operating characteristic (ROC) curves in Figure 3 for all the six databases in the lost-key scenario, with the key length of v and w set to n=149. The MCC-based real-valued, fixed-length feature vector T in (1) is of length m=299, so n=149 is the maximum allowed length to satisfy $1<n\leq \frac{m}{2}$. We observe from Figure 3 that among the six databases, the proposed authentication system shows the best performance over database FVC2002 DB1 and the worst performance over database FVC2004 DB2. Such performance is attributed to the high quality of fingerprint images in FVC2002 DB1 and the poor quality of fingerprint images in FVC2004 DB2.

**Figure 4:**
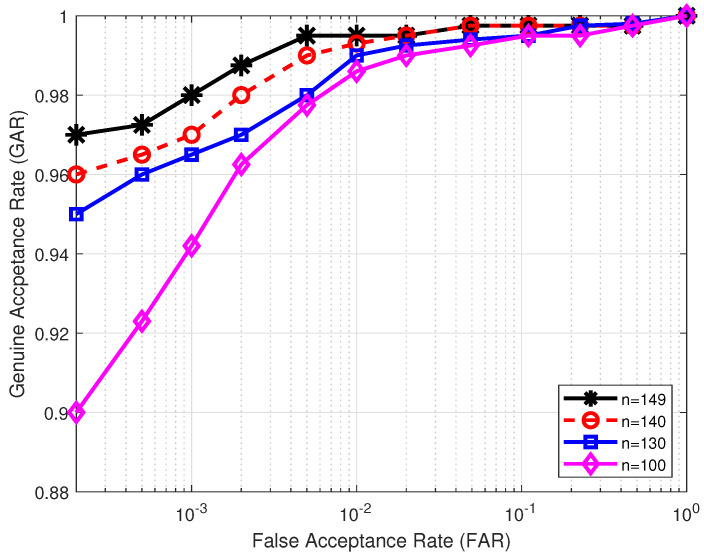
ROC curves for different key lengths evaluated over database FVC2002 DB2 in the lost-key scenario under the original FVC protocol.

Next, we evaluate how the key length affects the performance of the proposed authentication system. In our cancelable template design, there are two randomly generated user-specific keys v and w, both of length *n*. User key v is responsible for extracting elements from the original MCC-based real-valued, fixed-length feature vector T so as to form the vector x in (4). User key w is in charge of extracting elements from vector $\bar{x}$ in (5) to produce vector y in (6). To assess the impact of key length *n* on the performance of the proposed system, we vary the value of *n* from large to small. Table 2 illustrates the effect of different key lengths on the matching performance in comparison with the baseline, that is, the unprotected real-valued MCC-based features with fixed-length m=299. It can be seen from Table 2 that for all the databases, EER worsens when the key length *n* decreases. This is because a small value of *n* makes the original MCC-based feature vector T contribute less to the resultant cancelable template than a large value of *n*. Figure 4 shows the ROC over database FVC2002 DB2 for different key lengths.

Then, we compare the performance of the proposed authentication system for key length n=149 with that of the state-of-the-art cancelable fingerprint templates. This performance comparison is reported in Table 3. It is clear from Table 3 that the proposed system exhibits competitive performance over all six databases, and in particular demonstrates superior performance over databases FVC2002 DB1-DB3 and FVC2004 DB2 and DB3. The strong performance of the proposed method is attributed to our cancelable template design (see Section 3.2), which, by and large, maintains the good performance of the baseline (i.e., the unprotected fixed-length real-valued MCC-based features), as shown in Table 2 when n=149, but our method also plays the role of template protection.

### 4.2. Analysis of Computational Cost and Template Size

In this section, we investigate the computational cost and template size of the proposed authentication system. Table 4 lists the average time of generating the cancelable template as well as the average matching time using the designed cancelable template and the original MCC-based real-valued, fixed-length feature vector T. We can see from Table 4 that the average matching time using the designed cancelable template is much less than that using the original MCC-based feature vector (i.e., the baseline). The savings in matching time come from the reduced template size, because the proposed system shortens the length of the cancelable template by at least 50%, as $1<n\leq \frac{m}{2}$. Furthermore, Table 5 compares the average time of cancelable template generation of the proposed system with that of the state-of-the-art cancelable template methods. Clearly, the processing speed of the proposed system over all six databases is the fastest among all the methods in Table 5. The results in Table 4 and Table 5 are obtained by running MATLAB 2021a on a computer with a 3.41 GHz Intel (R) Core (TM) i7-6700 CPU.

Table 6 reports the template size of the proposed system in comparison with the state-of-the-art cancelable template methods. Clearly, the proposed system has the smallest template size among all the methods in Table 6.

### 4.3. Revocability and Diversity

**Figure 5:**
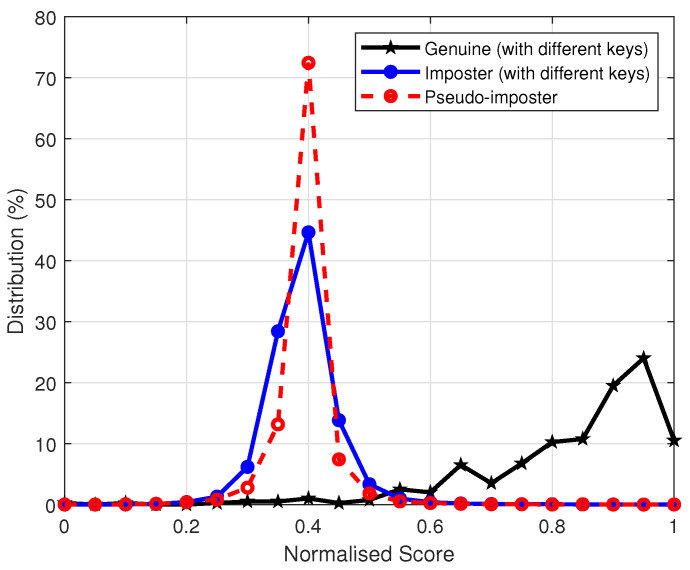
Genuine, Pseudo-imposter and imposter distributions over FVC2002 DB2.

In this section, we evaluate the revocability and diversity of the proposed authentication system. Revocability and diversity are considered important properties of cancelable biometrics, which require that if a stored template is compromised, it can be replaced with a new template by altering the user-specific key. The new template and the compromised template should be completely unrelated. To examine the revocability and diversity of the proposed system, we produced 100 transformed templates from the first impression of each finger in database FVC2002 DB2 by randomly generating different user keys v and w. These pseudo-imposter templates were matched with the original ones. The genuine, pseudo-imposter and imposter distributions are plotted in Figure 5, which shows that the pseudo-imposter and imposter distributions largely overlap. Their mean and standard derivation are very close to each other, given by the mean 0.3902 (pseudo-imposter) and 0.3913 (imposter), and standard derivation 0.055 (pseudo-imposter) and 0.053 (imposter).

### 4.4. Unlinkability

Unlinkability is another essential property of cancelable biometrics. It guarantees the privacy of biometric data and prevents cross-matching when users are registered in different applications using the same biometrics. Based on the framework presented in [28], we assess the unlinkability of the proposed authentication system by determining mated and non-mated sample score distributions at both local and global levels. Mated sample scores are obtained by comparing templates produced from the same impression of a finger using different keys, while non-mated scores are computed by comparing templates generated from different fingers using different keys.

Two unlinkability measures, score-wise linkability $D_{↔}\left(s\right)$ and system overall linkability $D_{↔}^{sys}$, are defined in [28]. The score-wise linkability $D_{↔}\left(s\right)$ determines the amount of linkability of protected templates for each matching score *s* of mated and non-mated sample score distributions. Thus, for a specific score *s*, $D_{↔}\left(s\right)=0$ means that two templates are fully unlinakble, while $D_{↔}\left(s\right)=1$ means that two templates are fully linkable. The intermediate values between 0 and 1 indicate the degree of linkability at specific matching scores. On the other hand, the system’s overall linkability $D_{↔}^{sys}\in \left[0,1\right]$ estimates the linkability of the entire template protection system, independent of matching scores. When $D_{↔}^{sys}=0$ (or $D_{↔}^{sys}=1$), the template protection system is fully unlinkable (or fully linkable).

**Figure 6:**
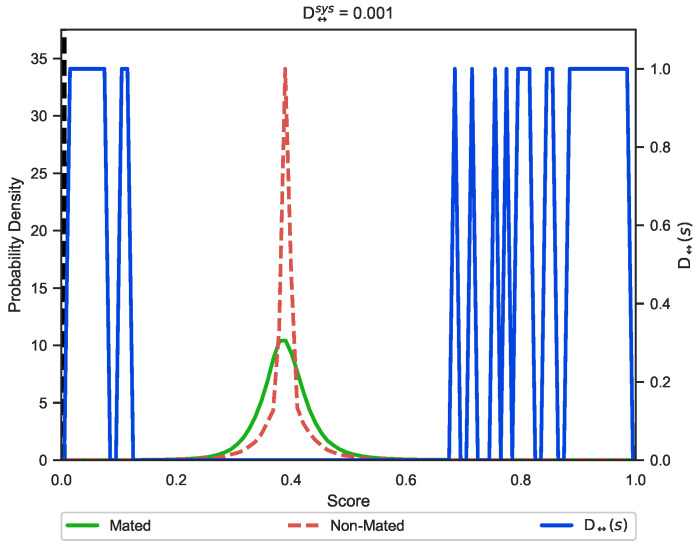
Unlinkability analysis of the proposed authentication system using mated and non-mated score distributions.

To access the unlinkability of the proposed authentication system, we produced 100 transformed templates of the first impression of each finger in database FVC2002 DB2 with random user keys v and w. We obtained the mated scores by comparing each transformed template with the 100 newly produced templates using different keys, giving 10,000 mated scores. We got the non-mated scores by comparing the transformed template of each finger with all other different fingers, yielding 4950 non-mated scores. Figure 6 shows the mated and non-mated sample score distributions—they mostly overlap. As $D_{↔}^{sys}=0.001$, the proposed authentication system is almost fully unlinkable.

### 4.5. Security Analysis

In this section, we conduct the security analysis of the proposed authentication system. We first analyze non-invertibility of the proposed method and then examine whether revoked template attacks and masquerade attacks can be defied.

#### 4.5.1. Non-Invertibility Analysis

In the context of cancelable biometrics, non-invertibility refers to the computational infeasibility of retrieving original biometric features from a compromised cancelable template. It is important for secure biometric authentication systems to possess non-invertibility as this property ensures the security of raw biometric data.

We analyze the non-invertibility of the proposed system in the worst-case scenario, where an adversary manages to acquire all possible information, namely the transformed template z and user keys v and w. Since v and w are only index vectors, even though they are compromised, the adversary is unable to know the specific values of the elements in vectors x and y whose indices match entries in v and w, respectively. Moreover, the proposed transformation constitutes a many-to-one mapping, as it follows from (7) that there exist infinitely many possible vectors x and y that can render the same z. Thus, it is computationally infeasible to restore x and y from the transformed template z and index vectors v and w.

#### 4.5.2. Revoked Template Attacks

We evaluate if the proposed authentication system can defend attacks when an adversary uses a revoked template. We consider the following two attack scenarios [29]:

- Type-I Attack: A revoked template is employed to attack a system containing a renewed template produced from the same impression.
- Type-II Attack: A revoked template is employed to attack a system containing a renewed template produced from another impression of the same finger.

Two different levels of security are assessed in each attack scenario [29] over database FVC2002 DB2:

1. Medium security: the matching threshold is set to 0.1% FAR.
2. High security: the matching threshold is set to 0% FAR.

A total of 500 (=5×100) Type-I attacks and 1000 (=(5×4)/2)×100) Type-II attacks were launched. Table 7 reports the percentage of successful attacks at medium and high security levels. It can be seen from Table 7 that the proposed authentication system can combat revoked template attacks.

#### 4.5.3. Masquerade Attacks

Masquerade attacks are similarity-based attacks where an adversary fakes a synthetic input that is very similar to the actual template. We simulated the masquerade attack by fabricating real-valued input vectors which resemble the original MCC-based feature vector T. The faked input of length 299 was generated by randomly choosing a small portion of elements (e.g., 10 out of 299 elements) from the original feature vector T and replacing them with values close to the original ones. Hence, the fake input shares a large degree of similarity with T.

We evaluate whether the proposed authentication system can tackle masquerade attacks in Type-I and Type-II attack scenarios at medium and high security levels using database FVC2002 DB2. The percentage of successful masquerade attacks is given in Table 8, where it is shown that the proposed system is able to defend masquerade attacks.

## 5. Conclusions and Future Work

In this paper, we proposed a secure online fingerprint authentication system. Designed to take advantage of the desired features of 5G networks, the proposed authentication system can be applied to protecting data security in the IIoT. The proposed authentication system has the functionality of template protection, empowered by the designed cancelable fingerprint template, which provides strong security to user privacy and critical data. The transformed (protected) template is transmitted over the 5G network so that fast online matching is conducted in the cloud server. Moreover, the proposed system has a size-reduced template compared to the baseline, unprotected system, thus saving computational costs and benefiting IIoT devices. In addition, the extensive experiment results on six public fingerprint databases show the favorable performance of the proposed authentication system, manifested by the low EER, when compared with state-of-the-art cancelable fingerprint templates.

As for future work, since the quality of minutia-based local features plays an important role in authentication performance, we will continue to develop discriminative and robust feature representations. In addition, we will put more effort into the design of secure and lightweight biometric authentication systems that suit the IoT/IIoT environment.

## Figures and Tables

**Table 1:** Comparison of state-of-the-art cancelable fingerprint templates.

Method	Feature Transformation	Advantage	Disadvantage
[12]	Random projection	Good authentication performance on high-quality datasets	Unsatisfactory authentication performance on low-quality datasets
[13]	Window-shift-XOR and partial discrete wavelet transform	Strong security	Large template size
[14]	Dyno-key model	Strong security	Limited recognition accuracy over low-quality datasets
[15]	Index-of-Max (IoM) hashing	Able to overcome intra-class variations	(1) Expensive computations due to considerable amounts of hash functions. (2) Similarity-based attacks can partially break the irreversibility of the method.
[17]	Sparse IoM (SC-IoM)	Meets revocability and unlinkability requirements	Expensive computations due to considerable amounts of hash functions.
[18]	Fourier-Mellin transform	Strong security	(1) Unsatisfactory performance on low-quality datasets. (2) Slow computational time.
[19]	Indexing-Min-Max (IMM) hashing	Good authentication performance	Slow processing time
[20]	Minimum hash signature and extended feature vector	Good privacy for the storage of user information	Inadequate security analysis
[21]	One permutation hashing	(1) Good authentication performance. (2) High efficiency.	Slow computational time
[22]	Extended feature vector (EFV)	Meets revocability and unlinkability requirements	(1) Limited performance evaluation on low-quality datasets. (2) Large template size.
[23]	Linear convolution	Meets revocability and linkability requirements	Limited performance evaluation on low-quality datasets

**Table 2:** EER (%) of the proposed system when the key length varies.

Key Length *n*	FVC2002			FVC2004		
	DB1	DB2	DB3	DB1	DB2	DB3
Unprotected real-valued MCC-based features of fixed-length 299	0	0.41	0.69	2.30	3.09	1.69
n=149	0.04	0.50	0.99	2.77	3.28	1.75
n=140	0.14	0.60	1.03	2.96	3.51	2.02
n=130	0.24	0.81	1.22	3.07	4.01	2.32
n=100	0.48	1.07	2.02	3.50	4.24	3.29

**Table 3:** EER (%) comparison between the proposed system and the state-of-the-art cancelable fingerprint templates in the lost-key scenario under the original FVC protocol.

Cancelable Fingerprint Template Design	FVC2002			FVC2004		
	DB1	DB2	DB3	DB1	DB2	DB3
Shahzad et al. [13]	1.57	1.50	4.93	10.49	8.62	-
Bedari et al. [14]	1.38	1.35	4.21	8.89	7.63	-
Jin et al. [15]	0.22	0.47	3.07	4.74	6.85	-
Kim et al. [17]	0.55	0.93	-	5.81	4.10	3.99
Abdullahi et al. [18]	0.36	0.54	2.40	2.35	5.93	2.37
Li et al. [21]	0.19	0.51	3.44	**1.49**	3.80	4.15
Lee et al. [22]	0.30	0.56	-	2.42	6.27	-
Yang et al. [23]	1.75	1.39	4.11	-	7.75	-
Proposed method (*n* = 149)	**0.04**	**0.50**	**0.99**	2.77	**3.28**	**1.75**

**Table 4:** Average time ($\times 10^{-5}$ s) for cancelable template generation and fingerprint matching.

Average Time	FVC2002			FVC2004		
	DB1	DB2	DB3	DB1	DB2	DB3
Cancelable template generation (*n* = 149)	5.2303	5.1454	5.4253	5.1722	5.2168	5.3609
Matching using the designed cancelable template	0.5562	0.5037	0.5593	0.5194	0.5112	0.5328
Matching using the original feature vector T (*m* = 299)	1.4840	1.4276	1.4880	1.8768	2.0215	1.5022

**Table 5:** Comparison of the cancelable template generation time (in second) between the proposed system and the state-of-the-art cancelable fingerprint templates.

Average Time	FVC2002			FVC2004		
	DB1	DB2	DB3	DB1	DB2	DB3
Bedari et al. [14]	0.0398	0.0491	0.0267	0.0444	0.0387	-
Jin et al. [15]	0.0072	0.0075	0.0072	0.0072	0.0074	0.0070
Abdullahi et al. [18]	0.0763	0.0362	0.0925	0.0291	0.4651	0.1240
Lee et al. [22]	0.01545	0.01539	-	0.01592	0.01481	-
Proposed method (*n* = 149)	**0.000052**	**0.000051**	**0.000054**	**0.000052**	**0.000052**	**0.000053**

**Table 6:** Comparison of the template size (bits) between the proposed system and the state-of-the-art cancelable fingerprint templates. Symbol *K* represents the number of minutiae in a fingerprint image.

Cancelable Template Methods	Cancelable Template Size (Bits)
Shahzad et al. [13]	73,728×K
Bedari et al. [14]	1036×K
Abdullahi et al. [18]	2188×K
Jin et al. [15]	19,200
Proposed method	9536

**Table 7:** Percentage of successful revoked template attacks at medium and high security levels.

Security Level	Type-I Attack	Type-II Attack
Medium security	0.2%	0.1%
High security	0%	0%

**Table 8:** Percentage of successful masquerade attacks at medium and high security levels.

Number of Elements Changed in T	Medium Security		High Security	
	Type-I Attack	Type-II Attack	Type-I Attack	Type-II Attack
10	0.2%	0.1%	0%	0%
20	0.2%	0.1%	0%	0%
30	0.2%	0.2%	0%	0%

## Data Availability

Not applicable.
